# Complement-Opsonized HIV Modulates Pathways Involved in Infection of Cervical Mucosal Tissues: A Transcriptomic and Proteomic Study

**DOI:** 10.3389/fimmu.2021.625649

**Published:** 2021-05-20

**Authors:** Cecilia Svanberg, Rada Ellegård, Elisa Crisci, Mohammad Khalid, Ninnie Borendal Wodlin, Maria Svenvik, Sofia Nyström, Kenzie Birse, Adam Burgener, Esaki M. Shankar, Marie Larsson

**Affiliations:** ^1^ Division of Molecular Medicine and Virology, Department of Biomedicine and Clinical Sciences, Linköping University, Linköping, Sweden; ^2^ Department of Obstetrics and Gynecology, University Hospital, Linköping, Sweden; ^3^ Department of Obstetrics and Gynecology, Region Kalmar County, Kalmar, Sweden; ^4^ Department of Biomedical and Clinical Sciences, Linköping University, Linköping, Sweden; ^5^ Department of Clinical Immunology and Transfusion Medicine, and Department of Biomedical and Clinical Sciences, Linköping University, Linköping, Sweden; ^6^ National HIV and Retrovirology Labs, JC Wilt Infectious Disease Research Centre, Public Health Agency of Canada, Winnipeg, MB, Canada; ^7^ Center for Global Health and Diseases, School of Medicine, Case Western Reserve University, Cleveland, OH, United States; ^8^ Infection Biology, Department of Life Sciences, Central University of Tamil Nadu, Thiruvarur, India

**Keywords:** HIV - human immunodeficiency virus, innate immunity, complement opsonized HIV-1, transcriptomics, proteomics, cervical tissue, primary infection

## Abstract

Genital mucosal transmission is the most common route of HIV spread. The initial responses triggered at the site of viral entry are reportedly affected by host factors, especially complement components present at the site, and this will have profound consequences on the outcome and pathogenesis of HIV infection. We studied the initial events associated with host-pathogen interactions by exposing cervical biopsies to free or complement-opsonized HIV. Opsonization resulted in higher rates of HIV acquisition/infection in mucosal tissues and emigrating dendritic cells. Transcriptomic and proteomic data showed a significantly more pathways and higher expression of genes and proteins associated with viral replication and pathways involved in different aspects of viral infection including interferon signaling, cytokine profile and dendritic cell maturation for the opsonized HIV. Moreover, the proteomics data indicate a general suppression by the HIV exposure. This clearly suggests that HIV opsonization alters the initial signaling pathways in the cervical mucosa in a manner that promotes viral establishment and infection. Our findings provide a foundation for further studies of the role these early HIV induced events play in HIV pathogenesis.

## Introduction

The most common mode of HIV-1 spread is through heterosexual intercourse, and a vast majority of new infections globally occur in females. Immunobiological investigations of HIV infection involving the cervical mucosa are of paramount importance, especially to better understand the events associated with initial infection as well as viral dissemination in the host. To attain successful establishment of infection in the host, HIV virions need to cross the genital mucosal barrier and/or encounter certain immune cells that can transfer the viruses to target cells in the epithelium (1, 2). The target tissue for initial viral invasion appears to be the simple columnar and the stratified squamous epithelia of the endocervix and ectocervix, respectively (2). Evidence suggests that 24h following exposure, dendritic cells (DCs) with captured virions reach the draining lymph nodes where they transfer infectious HIV to bystander cells (2, 3). The primary target cells of HIV and productive viral infection appear to be tissue-resident memory (TRM) CD4+ T cells (3).

The mucosal barrier has several defense mechanisms to deploy against diverse types of invading pathogens: the mucus as physical barrier and a multitude of antimicrobial factors that are produced both by epithelial cells as well as leukocytes to protect the host. Given that mucosal epithelial cells represent the front-line barrier for HIV to surpass before establishing an infection, the cells do play a paramount role in viral acquisition and dissemination (4). Besides, epithelial cells also produce several antimicrobial factors as well as regulate mucosal integrity toward molecules and microbes that translocate across the cellular layer. In addition, they also express pathogen recognition receptors (PRRs) that respond to pathogenic invasion by triggering the initiation of type I and type III interferon (IFN) responses. Such epithelial responses have been shown to restrict HIV replication in macrophages (4). Furthermore, HIV exposure also increases the production of inflammatory mediators such as TNF by epithelial cells (5). Secreted antimicrobial factors include α- and β-defensins and lysozymes are part of the mucosal defense (6). In addition to secreted factors, local microbiota and underlying infections also appear to impact the mucosal defense and prevent the initial binding of HIV. Inflammatory conditions of the vagina, such as bacterial vaginosis, are associated with increased risk of HIV acquisition (7, 8). Pre-existing sexually transmitted infections, such as herpes simplex virus type 2 (HSV-2), enhance the risk of HIV acquisition due to recruitment of more target cells into the genital mucosa (9). Furthermore, HSV-2 modulates the mucosal immune cells to be more receptive to HIV infection (10, 11). The presence of pro-inflammatory cytokines produced during infection and inflammation, especially TNF, is associated with enhanced HIV infection (12, 13). Interestingly, the levels of type I IFN can have pleotropic effects depending on the circumstances, and have been shown to increase both uptake and dissemination of HIV as well as limiting viral replication and protecting against HIV acquisition (14). Other factors that are likely to influence the initial mucosal responses to HIV and other pathogens include semen, which has been shown to induce inflammation and increase HIV acquisition (15). The viral transmission fluid also contains factors that inhibit viral infection, such as antibodies, and several types of lectins (16).

The female genital compartment contains an array of soluble factors that participate in local innate immune defenses; notable among them are complement proteins such as C1q and C3. During the initial phases of infection, the transferred HIV virions are opsonized by complement proteins (17) such as inactivated C3b (iC3b) and C1q that are attached to the viral surface (18). The complement protein C3 when activated forms C3b that can be part of the complement cascade that destroys pathogens or infected cells by cytolysis and virolysis. Then again in the case of HIV, C3b is converted to iC3b by complement regulatory factors found in the viral envelope and thereby protecting the virus from virolysis (19). In addition, C1q adheres to gp120 and gp41 and this process appears to be particularly efficient in the presence of anti-gp41 antibodies (20). The opsonization pattern of the virions will be affected by the surrounding microenvironment and the type of tissue or body fluid (18) due to different levels of antibodies and complement factors as well as tissue/fluid specific factors. Virus particles that are transferred *via* mucosal transmission should be opsonized by complement and other factors present in cervical secretions or seminal fluids, including HIV specific antibodies if the donor is HIV-1 seropositive (18). We have previously shown that complement opsonization influences HIV infection by enhancing viral uptake by emigrating cervical mucosal DCs (21). Furthermore, complement opsonization of HIV dampens the inflammatory and antiviral responses of *in vitro* cultured immature DCs (22) and affects the pattern of chemotactic factors produced by the DCs, which in turn alters the migration of NK cells (23). In this light, it is critical to explore the effects of opsonization on the initial phases of HIV infection to advance our understanding of what factors that are important for the establishment infection.

The cervical mucosa consists of multiple layers of different tissue types, with the endocervix being lined with a single layer of columnar epithelium, a transformation zone where the columnar cells change into squamous cells, and the ectocervix lined with stratified squamous epithelial cells. The different regions of the female reproductive tract contain diverse subsets of DCs and T cells (24–27). DCs play a major role in the dissemination of HIV infection in the host (25). DC subsets are found both at the basal membrane as well as in the outer layers of the epithelium, and represent one of the first cell types to encounter HIV in the cervical mucosa (28). DCs and myeloid DCs in cervical mucosa play an important early role in the capture the HIV and harboring of infectious virions and in the transmission of HIV to CD4+ T cells (29, 30). For instant, the epithelium also contains CD1a+ DCs that reportedly preserve HIV virions and support their replication (31).

DCs are efficient in capturing, effectively preserving, and transporting the virus *via* the mucosa to infect T cells in the lymphoid organs (32). The CD4+ T cells in the cervix are located close to the basal membrane but can also be found within the stratified epithelia of the ectocervix, as well as within the columnar epithelia of the endocervix (33). It is also worth noting that the T helper (TH) 17 T cell subsets are more abundant in the ectocervix and endocervix than in the endometrium. Further, it has also been reported that endocervical CD4+ T cells are highly susceptible to HIV infection *in vitro*, whereas the same cell type present in the endometrium are relatively resistant (27). In addition to DCs and T cells, the cervical mucosa also contains macrophages and CD14+ myeloid cells; the latter being the most abundant immune cell type in the tissue (33).

It has been reported that the HIV transmitter/founder virions are foremost CCR5-tropic, and that a vast majority of the new infections is established by one single transmitted founder virus (34, 35). The ability to enter target cells is attributed to the HIV envelope (Env) protein that encompasses the gp120 and gp41 subunits, and notably, on the founder viruses the gp120 appears to have unique phenotypes (16, 36). During the initial phases of HIV infection, the viral population is markedly homogenous. After the initial phase, a rapid virus evolution occurs, likely attributed to the high replicative potential of HIV, leading to increased rates of genetic diversity, and hence a highly heterogenous HIV population in an infected individual (16, 34, 35).

The ex vivo HIV infection of cervical mucosa has been studied by several groups including ours (1, 2, 28, 31, 37, 38), but the initial immune responses of the human cervical mucosa to HIV exposure have not been well characterized. So, we endeavored to investigate the effects of either free or complement-opsonized HIV on the initial events during viral transmission, using a cervical explant model to get insights of factors that facilitate or protects against viral transmission that could be utilized for the development of effective protective vaccines/microbicides. We used RNA sequencing and proteomics to delineate the molecular events underlying the differences in gene and protein expression after exposure to the free and the complement-opsonized HIV. We found that the infection levels of the cervical mucosal tissue and emigrating immune cells were enhanced after exposure to complement-opsonized HIV and complement and antibody-opsonized HIV compared to free HIV. This increase in infection was mirrored in the biological function and disease pathway analyses of the transcriptome and proteome of the tissues, with multiple pathways supporting viral infection and replication significantly enriched/activated. Signaling pathways involved in immune activation were induced 6h post exposure, but this activation was downregulated at 24h post exposure, both at the transcript and protein levels indicating an active immune suppression induced by HIV. These early events occurring in the cervical tissue are most likely crucial for the establishment of the viral infection and for persistence.

## Materials and Methods

### Study Subjects and Ethics Approval

Healthy cervical tissue specimens (N=41) were obtained from individuals that underwent prolapse surgery or a hysterectomy for benign conditions at the Department of Gynecology, University Hospital, Linköping, Sweden, after securing informed consent prior to surgery. All participants had normal Pap smears prior to surgery and showed no clinical signs of cervical pathology at surgery. The study was approved by the Linköping University Ethical Review Board (Ethical permit EPN M206-06).

### *Ex Vivo* Cervix Tissue Preparation and Culture

**Figure 1:**
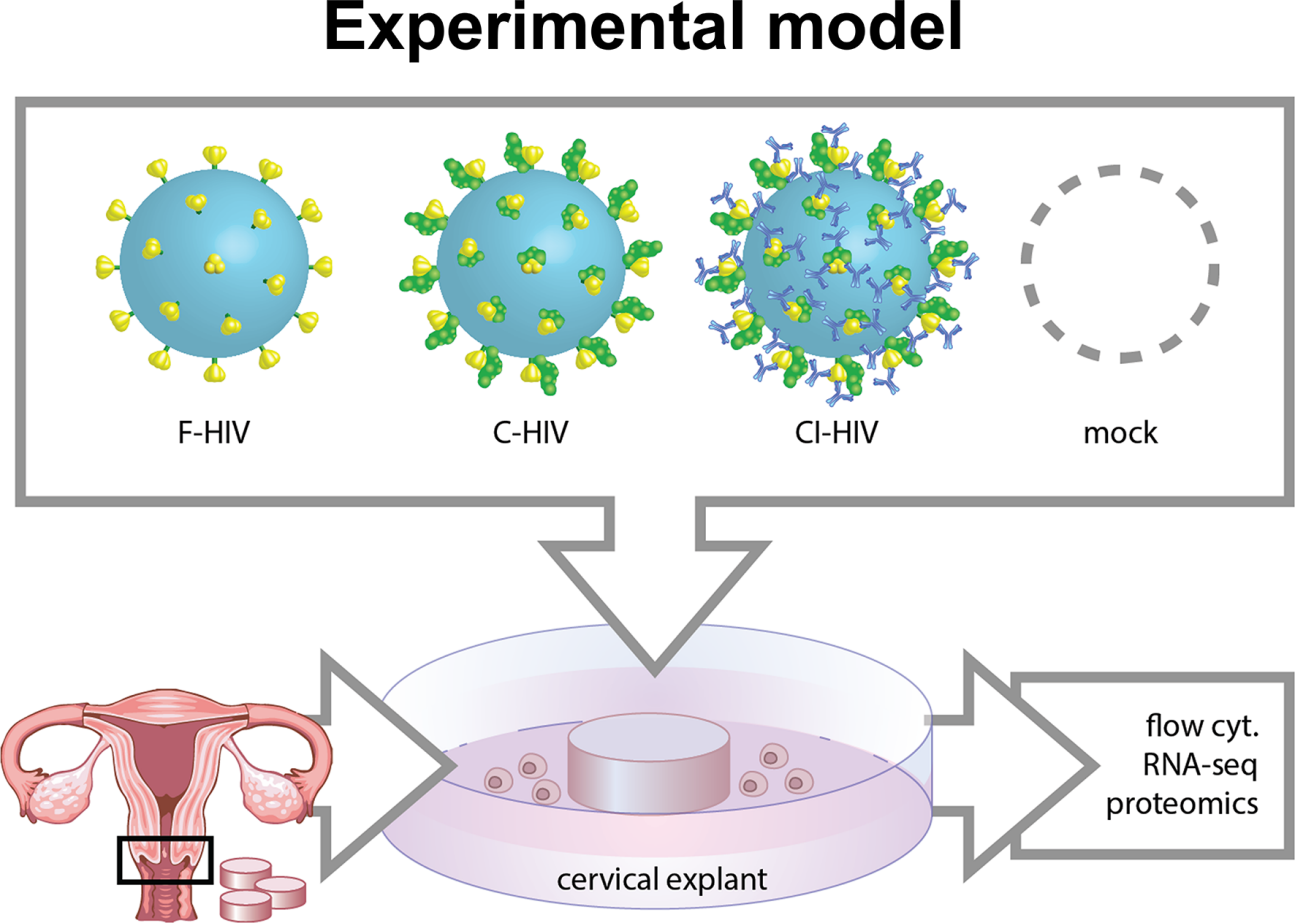
Model of HIV *in vitro* infection of cervical mucosal tissue. Cervical biopsies 3 mm^2^-8 mm^2^ cervical mucosal biopsies were spin-occulated with 250ng/ml opsonized referred to as free HIV-1 (F-HIV), complement-opsonized HIV-1 (C-HIV), complement and antibody opsonized HIV-1 (CI-HIV) or mock treatment for 2h. The tissues were thereafter washed and re-incubated, and then harvested after 6h, 24h, or 5 days. The mucosal tissues and emigrated mucosal cells were harvested and analyzed using RNA seq, proteomics, qPCR, and flow cytometry.

The cervical tissue biopsies collected from the study participants were kept on ice and processed within 30 min after resection. The cervical tissues were punched into 3 or 8 mm^2^ biopsies (punch-biopsy). Thereafter, the epithelial layer and lamina propria were separated from the underlying stroma using sterile surgical scissors. Ectocervical and endocervical biopsies were kept separated and placed in a culture media that contained RPMI-1640, 20μg/mL Gentamicin, 10mM HEPES (Fisher Scientific, Gothenburg, Sweden), 5% pooled human serum (Novakemi, Sollentuna, Sweden) and 2.5μg/mL fungizone (Sigma-Aldrich) in such a way that the epithelial layer was in line with the liquid interface. Subsequently, the cervical tissues were spin inoculated at 1200g together with HIV-1 BaL (250 ng/mL) for 2h at 37°C. Following infection, the explants were washed using RPMI, moved into a 6-well-plate that contained 1mL of culture media and were left in the incubator (37°C, 5%CO_2_) for 6h up to 5 days before explant and/or emigrating cells were collected for further investigations. Culture supernatants were collected for ELISA/CBA analysis of cytokines and HIV-1 p24 assays. For graphical illustration on how the procedure was performed see **Figure 1**.

### Virus Production and Opsonization

HIV-BaL SUPT1-CCR5 CL.30 (Lot #P4238, Lot #4213, Lot #4336, and Lot #4369) Biological Products Core/AIDS and Cancer Virus Program, Frederick National Laboratory, Frederick, MD, USA was produced using chronically infected cultures of the ACVP/BCP cell line (No. 204), originally derived by infecting SUPT1-CCR5 CL.30 cells (generously gifted by Dr. J. Hoxie, University of Pennsylvania) with an infectious stock of HIV-1BaL (NIH AIDS Research and Reference Reagent Program, Catalog No. 416, Lot No. 59155). Virus was purified and concentrated as previously described (39) and aliquots were frozen down. All virus preparations were assayed for infectivity. In addition, HIV-1 THRO founder virus (Lot# 4393) produced in A66-R5 CL.29 cells, were used in a few experiments. Virus was purified and harvested using flow centrifugation for 30 min, diluted to <20% sucrose, pelleted at 100,000×g for 1h and frozen in liquid N2 vapor. HIV was used in different forms, viz., Free HIV (F-HIV, RPMI only), complement-opsonized HIV (C-HIV) and complement and antibody opsonized HIV (CI-HIV). C-HIV was achieved by incubating HIV with 25-50% single donor human serum for 1h at 37°C. CI-HIV was achieved by incubating HIV with 25-50% single donor human serum and a mixture of human gamma globulin non-specific 20mg/mL (Octagam, Stockholm, Sweden) and 0.2mg/mL HIV specific neutralizing IgG (HVIGLOB (40), a kind gift from Prof. Jorma Hinkula, Linkoping University, Sweden) for 1h at 37°C. Verification of the complement opsonization protocol was done by assessing the production iC3b by ELISA **(** **Supplementary Figure 1** **)**.

### Flow Cytometry

Emigrating cells were collected and stained with CD3-PE-Cy7 (clone UCHT1), CD4-PE (clone OKT4), CD1a-APC (clone HI149), CD1c-PerCp-Cy5.5 (clone F10/21A3) (BD Bioscience, Stockholm, Sweden), CD163-APC-Cy7 (clone GHI/61, Nordic Biosite) and Zombie Aqua™ Fixable Viability Kit (BioLegend). For intracellular HIV-1 staining, the cells were washed, fixed using 4% PFA (Fisher Scientific) and permeabilized using 0.2% saponin and 0.2% FBS in PBS before incubating with either anti-HIV-1 p24 FITC mAb (KC57, clone FH190-1-1, Beckman Coulter) or its corresponding isotype control (IgG1 mouse, Beckman Coulter). The cells were acquired on a BD FACSCanto II flow cytometer, and data were analyzed using FlowJo software (Treestar, Ashland, OR, USA).

### Quantitative LC-MS Proteomics and Multiplex Proteomics Immunoassay

Matched ectocervical biopsies (n=5, per treatment) were exposed to F-HIV, C-HIV or CI-HIV, and cultured for 24h. Fifty micrograms of each sample was digested with trypsin overnight, labeled with iTRAQ isobaric tags (8-plex) according to manufacturer’s protocols (AB Sciex), fractionated *via* reverse phase liquid chromatography and ran on a Velos Orbitrap mass spectrometer (Thermo Fisher) using LC-MS/MS as described previously (22, 41). Searches were performed against the Human SwissProt (2015) database using Mascot (Matrix Science). The data obtained was thereafter analyzed using Scaffold (Proteome Software). Protein and peptide identifications thresholds were set to the following: 99% protein identification, 80% peptide identification and a minimum of 2 unique peptides required. All samples were normalized to a reference sample containing a mix of all samples included in the study. Groups were thereafter further normalized to its respective untreated (mock) tissue. Pathway and biological function analysis were performed using Ingenuity Pathway Analysis (IPA) software (Qiagen).

Supernatants were taken from cervical cultures at 96h, mixed 1:1 with RIPA lysis buffer (Sigma Aldrich) and was analyzed with a Proseek 130 ^®^ multiplex proteomics immunoassay at OLINK Proteomics (Uppsala, Sweden), which uses a proximity extension assay technology (42, 43). Both the Immune Response panel (https://www.olink.com/content/uploads/2017/07/1051-v1.2-Immune-Response-Panel-Content_final.pdf) and the Inflammation panel (https://www.olink.com/content/uploads/2019/04/1029-v1.3-Inflammation-Panel-Content.pdf) panel were used for analysis, each detecting 92 unique proteins.

### RNA Seq of HIV Exposed Cervical Mucosal Tissue

Whole cervical tissues were harvested 6 and 24 h post infection and cells were lyzed using Trizol (Thermofisher, Stockholm, Sweden) and mechanical disruption at 25Hz for 15 min (Tissuelyser, Qiagen). The cell lysates were centrifuged at a maximum speed for 10 min followed by mixing the supernatants with chloroform. After an additional centrifugation, the aquatic phase containing RNA was harvested and further purified using Isolate II RNA Mini or Micro Kit (Bioline, UK). An amount of 5ng of RNA was amplified using NuGEN’s Ovation RNA-Seq V2 kit (San Carlos, CA, USA) according to the manufacturer’s instructions. In short, cDNA was amplified from total RNA using a single primer isothermal amplification (SPIA) process. Later, the cDNA was purified using a MinElute Reaction Cleanup Kit (Qiagen). The cDNA samples were fragmented, blunt ended, ligated to barcoded adaptors, and once again amplified using the Ultralow System V2 kit (NuGEN) according to the manufacturer’s instructions. Final library size distribution was determined using an Agilent Bioanalyzer 2100. Libraries of both endocervix and ectocervix were sequenced on an Illumina NextSeq500 platform (San Diego, CA, USA). The sequencing files were quality checked using the FastQC and MultiQC programs, trimmomatic was used to remove the adaptors and to find low-quality bases. Reads were then mapped to the human reference genome hg19 using STAR. Counts for gene expression was determined using FeatureCounts. The counts were normalized and further analyzed for differentially expressed genes using R/DeSeq2. Analysis of pathways was done by Ingenuity Pathway Analysis (Qiagen), R analysis, Gene Ontology (GO) Enrichment Analysis (Geneontology.org), and *via* custom gene lists.

### RT-qPCR

RNA was extracted and purified as described above. cDNA synthesis was thereafter performed using SuperScript III Reverse Transcriptase First Strand cDNA Synthesis kit (Invitrogen, Carlsbad, CA, USA). mRNA was quantified using the SensiFAST SYBR^®^ Hi-ROX Kit (Bioline, UK) and CFX96 Touch Real-Time system (BIO-RAD Inc.). For all samples β-actin and GAPDH were used as housekeeping genes, and to compensate for variations between PCR runs, the values were further normalized as described previously by Rieu and Powers (44).

### Assessment of Cervical Productive Infection by HIV-1 p24 Gag ELISA and CBA

For HIV-1 p24 gag measurement, supernatants were treated with 0.5% Triton X-100 and the amount of p24 gag was determined by an *in-house* HIV-1 p24 capture ELISA using anti-p24 antibodies (kindly gifted by Prof. Jorma Hinkula). Cytometric bead arrays (BD Biosciences) were used to measure the levels of cytokines and chemokines in cell culture supernatants according to the manufacturer’s protocols.

### Statistical Analysis

GraphPad Prism 8 (GraphPad Software, La Jolla, CA) was used for data stratification and statistical analysis of PT-qPCR and ELISA and the data groups were compared using One-way ANOVA and Tukey’s post Hoc test (a two-sided paired t-test), p value <0.05 was considered statistically significant. RNAseq was analyzed using DESEQ2, which compensate for multiple comparisons/repeated measurements). Proteomic data analysis was performed in R (v3.6.2) using repeated measured Friedman test. Ingenuity pathway analysis parameters: A p-value cut-off of 0.05 was set as significantly affected molecules for both proteomics and transcriptomics data. IPA core analysis was used to identify which canonical pathways, disease and biofunctions as well as up-stream regulators that were either up- or down-regulated using Z-scores calculated by the program. Comparison analyses were used to compare pathways affected by each treatment arm. Heatmaps were used for visualization of data, and in some cases individual pathways was selected and molecules plotted separately for visualization of included molecules.

## Results

### Complement Opsonization of HIV Increases the Overall Infection of Cervical Mucosa and Infection of Emigrating DCs but Decreases Infection of Emigrating T Cells

**Figure 2:**
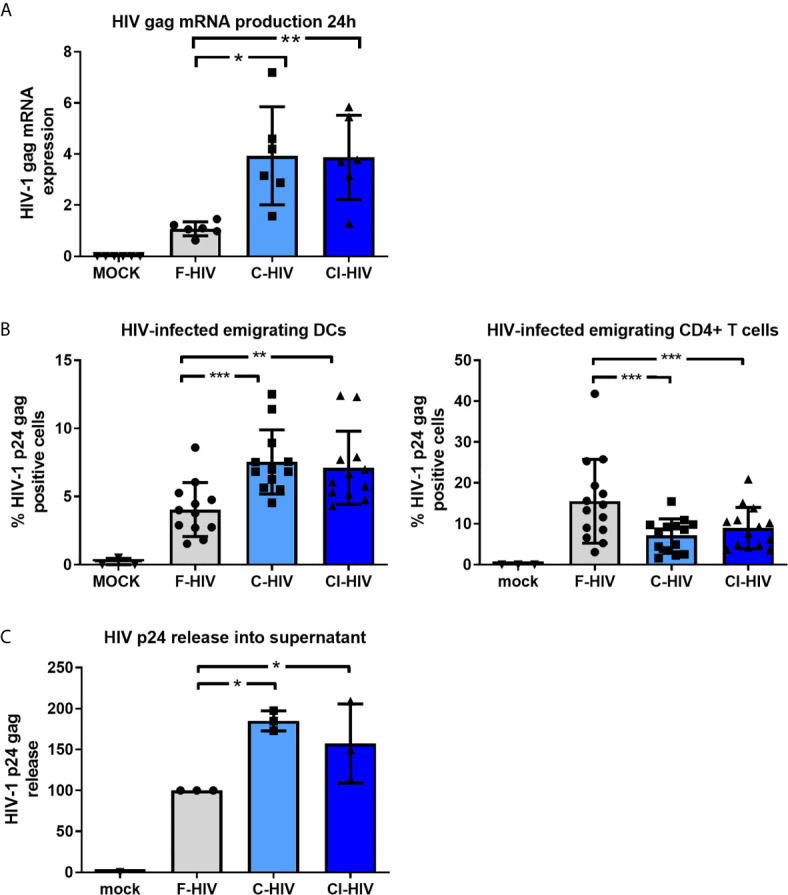
Complement opsonization of HIV increases infection of emigrating DCs but decreases infection of emigrating T cells. Cervical mucosal tissues were exposed to 250ng/ml HIV-1 BaL that was either F-HIV, C-HIV, or CI-HIV or mock-treated and cultured for 24h or 4-5 days. **(A)** The HIV-1 exposed cervical mucosal tissues were lysed, and RNA purified and used for qPCR to measure HIV1 mRNA (gag). **(B)** The emigrating cells were collected day 4 or 5 and the cells stained for anti-CD3, anti-CD4, anti-CD1a/CD1c. The cells, DCs (CD1a+) and T helper cells (CD3+CD4+), were thereafter stained for HIV p24 gag and analyzed using flow cytometry for the level of HIV infection (p24+). **(C)** The supernatants from the HIV-1 exposed cervical mucosal tissues were harvested day 4or 5 and analyzed for HIV-1 p24 gag in an ELISA (N=21). *p < 0.05, **p < 0.01 and ***p < 0.001.

The cervical biopsies were exposed to mock, F-HIV, C-HIV and CI-HIV and cultured between 6h to 5 days. After 24h of viral exposure, we measured the initial level of HIV-1 gag mRNA transcripts in the tissue by q-PCR. The complement-opsonized HIVs, both C-HIV and CI-HIV, induced a higher level of HIV gag mRNA transcripts in the mucosal tissue (**Figure 2A** **)**. To assess HIV-1 infection of the mucosal immune cells known to be involved in initial infection (2), i.e. DCs (45) and T cells (37), the emigrating cells were collected after 3-5 days of infection followed by flow cytometric investigation to determine the amount of HIV infected (p24 gag+) DCs (CD1a+ or CD1c+), and T cells (CD3+CD4+). Shown here are the cervical tissues infected for 4-5 days **(** **Figure 2B** **)**. Complement-opsonization entailed a higher infection of the emigrating DCs but induced lower infection of emigrating T cells compared to F-HIV **(** **Figure 2B** **)**. These findings are in line with what we have shown previously (21). The level of HIV-1 p24 gag released into the supernatant was measured on day 4-5 by an *in-house* HIV-1 p24 gag ELISA to assess the overall productive infection of the cervical biopsies. Infection was clearly enhanced in the C-HIV- and CI-HIV-infected cervical biopsies compared to F-HIV **(** **Figure 2C** **)**. These data demonstrate that opsonization represents a strategy employed by the virus to create an environment supporting enhanced infection of the cervical tissue. Next, to establish the effects HIV exposure had on cervical mucosal tissue and elucidate the underlying pathways and factors responsible for enhanced HIV infection induced by complement opsonization, we performed RNA sequencing to explore the transcriptome profiles and LS-MS proteomics to unveil the protein profiles.

### Complement Opsonization of HIV Alters Anti-Viral Innate Immune Signaling and Affects Key Inflammatory Regulators in the Cervical Mucosa

**Figure 3:**
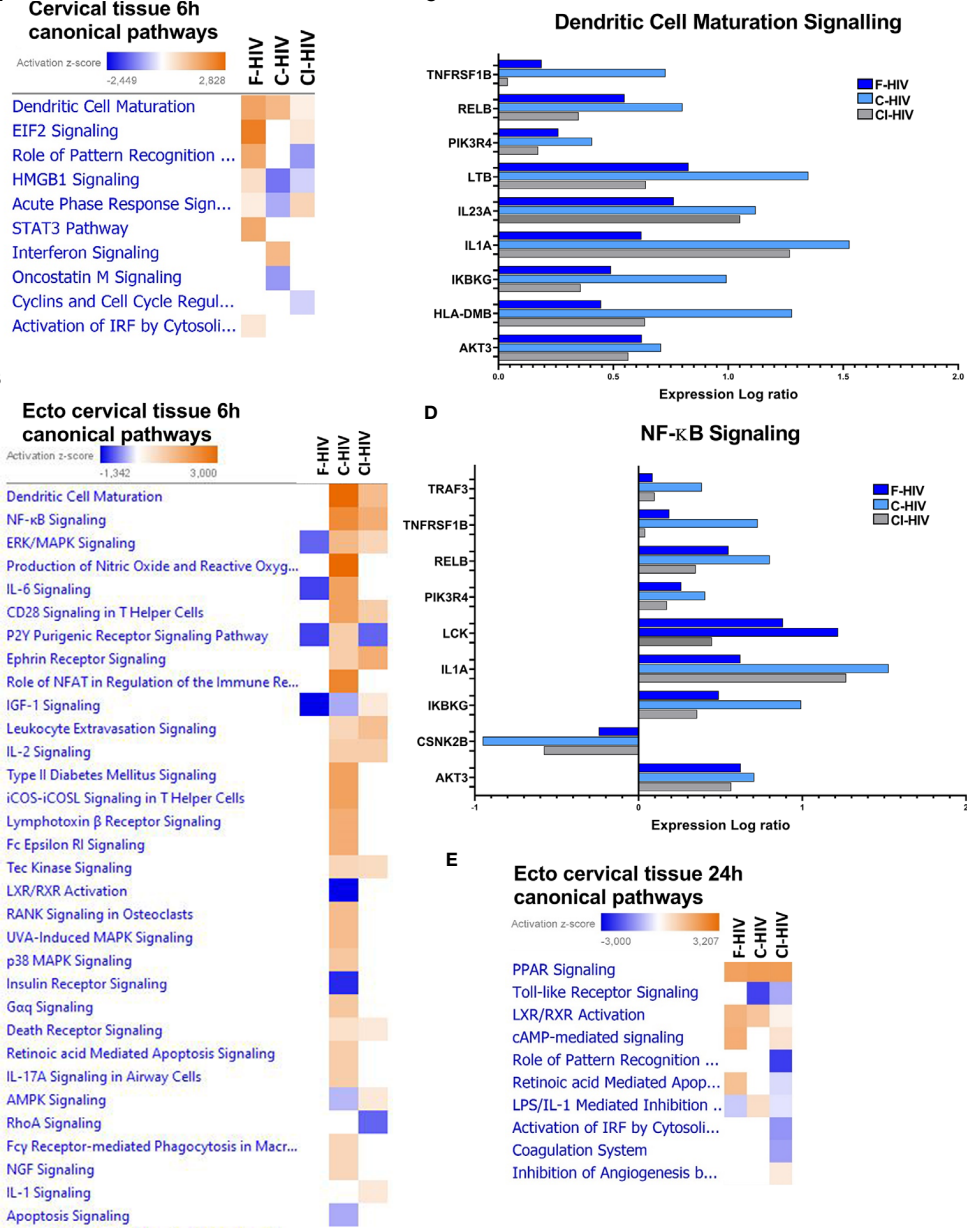
Complement opsonization of HIV alters innate immune signaling and affects key inflammatory regulators in cervical mucosa. Cervical mucosal biopsies (3mm) were spin-occulated with 250ng/ml F-HIV, C-HIV; CI-HIV or mock for 2 hours. The biopsies were then washed and incubated for an additional 4h or 22h. The tissues and emigrated cells were harvested, and RNA was purified and whole transcriptome sequencing was performed. **(A)** Canonical pathways affected by the HIV exposure in the complete, i.e. endo and ecto, cervical tissue at 6h. **(B)** Canonical pathways affected by the HIV exposure in the ecto cervical tissue at 6h. **(C)** Analysis of expression log ratio of factors from the canonical pathway, “Dendritic Cell Maturation” identified in ectocervix tissue only. **(D)** Analysis of expression log ratio of factors from canonical pathway” NF-kB Signaling” identified in ectocervix tissue only. **(E)** Canonical pathways affected by the HIV exposure in the ectocervical tissue only at 24h. (N=6). Heat maps display pathways significantly affected by a treatment arm (p < 0.05) and activation z-scores are displayed.

We set out to investigate the initial transcriptomic responses induced by HIV in the endocervical and ectocervical tissues exposed for 6h to mock, F-HIV, C-HIV or CI-HIV by RNA sequencing. The effects on the complete cervical tissue, i.e., the combined transcriptome of both endo- and ectocervical tissues were investigated (hereafter referred to as cervix tissue). The canonical pathway analysis showed that both C-HIV and CI-HIV altered the signaling pathways activated by HIV in the cervical tissue compared to F-HIV (**Figure 3A** **)**. The F-HIV activated a larger number of canonical pathways than C-HIV and CI-HIV at 6h. F-HIV-exposed mucosal tissue had significantly activated pathways (indicated by positive z-scores) such as “HMGB1 signaling”, “Role of pattern recognition receptors in recognition of bacteria and viruses”, “STAT3 pathway”, and “Activation of IRF by cytosolic pattern recognition receptors”. C-HIV had positive Z-score for interferon signaling **(** **Figure 3A** **)**. These pathways include pro-inflammatory cytokines such as HMGB1, CCL2 and CXCL8 (HMBG1 signaling pathway), IL6, IRF3, IRF7, CCL5 (Role of pattern recognition receptors in recognition of bacteria and viruses pathway), interferons type 1 and several interferon response factors and interferon stimulated genes (pathway “Activation of IRF by cytosolic pattern recognition receptors pathway). We used qPCR to confirm the dysregulated expression of the antiviral factors MXA and IFIT3 and proinflammatory factor IL6 and chemokine CXCL8 were more elevated in F-HIV compared to C-HIV and CI-HIV at 6h in cervix and at 24h had the IL6 and MX1 responses disappeared while the CXCL8 and IFIT3 responses remained **Supplementary Figures 2A, B**).

The canonical pathway called “Dendritic cell maturation” was activated in all groups, with significantly positive Z-score for the F-HIV and C-HIV exposed mucosal tissue groups, but not for CI-HIV **(** **Figure 3A** **)**. When restricting the analysis to the ectocervical tissue transcriptome, the canonical pathways “Dendritic cell Maturation”, “NF-κB Signaling”, “CD28 signaling in T helper cells” and “IL-2 Signaling” canonical pathway were all positive for C-HIV and CI-HIV, with the highest Z-score in the C-HIV group at 6h (**Figure 3B** **)**. This difference between the ectocervical tissue alone and complete cervical tissue regarding pathway such as “Dendritic cell Maturation” could be due to the tissue composition with more epithelial cell in the complete cervical tissue and/or immune cell composition and activation levels. The “Interferon signaling”, “cytokine profile”, “Dendritic cell maturation, “Production of nitric oxide and reactive oxygen species in macrophages”, “IL-6 signaling”, “Role of NFAT in regulation of the immune response” and “ICOS-ICOSL signaling in T helper cells” were some of the representative groups that were activated among C-HIV but not with F-HIV or CI-HIV **(** **Figure 3B** **)**. The overall effects on signaling pathways were higher for C-HIV-exposed mucosal tissues, whereas F-HIV generally induced negative, i.e., inhibition, Z-scores, if any, for the canonical pathways. When exploring the molecules involved in some of the different canonical pathways seen in ectocervical tissues, we found that C-HIV had a higher fold change than both F- and CI-HIV for almost all molecules assigned to the pathways “NFκB signaling” and “Dendritic Maturation Signaling” **(** **Figures 3C, D** **)**. Genes associated with both DC and T-cell activation were increased in ectocervical tissues exposed to C-HIV and CI-HIV whereas the F-HIV-exposed tissues remained unaltered by the virus. Most effects were seen after exposing the tissue to C-HIV. Genes such as LCK, WASP and AKT3 associated with T-helper cell functions (not shown) were affected as well as genes such as IL1A, IL23A and LTB (lymphotoxin beta) associated with Dendritic Cell Maturation Signaling and/or “NFκB signaling” were more highly upregulated in tissues exposed to C-HIV **(** **Figures 3C, D** **)**.

### The Transcriptome Profile Was Drastically Altered at the 24h Time-Point Compared to the Initial Responses Seen at 6h Exposure

In addition to the early 6h time-point, we explored the transcriptome profiles of the ectocervical tissues after 24h of HIV exposure. The canonical pathway analysis clearly demonstrated a decrease in the number of genes that were activated compared to the 6h time-point **(** **Figures 3A, E** **)**, fitting to previous findings where HIV induced a transient activation of the immune system (22, 46). Furthermore, the canonical pathways documented at 24h differed from the shorter 6h period of HIV exposure **(** **Figures 3A, E** **)**. The canonical pathway “cAMP mediated signaling” with genes such as AMP, ATP, ERK1/2 was significantly increased in F-HIV, C-HIV and CI-HIV compared to mock. In addition, the nuclear receptor signaling was evident with “PPAR signaling” and “LXR/RXR activation” having positive significant Z-score in all HIV groups (**Figure 3E** **)**. LXR/RXR activation had the highest positive Z-scores for F-HIV and genes in these pathways include ABCA1, ABCG1, APOA1, APOA4, APOC1, APOE, CCL2, CYP51A1, CYP7A1, HDL, HMGCR, IRF3 and LDL. Retinoic acid-mediated apoptosis signaling was found in F-HIV but not in C-HIV and CI-HIV (**Figure 3E** **)**. The metabolomic changes in the tissues induced by LXR/RXR signaling are induced at the 24h time-point but not as early as 6h. In summary, at 24h the transcriptome profiles of the cervical tissue displayed upregulation/downregulation of the second messenger cAMP, and nuclear receptors LXR/RXR pathways, the previous known to be involved in, and upregulated during HIV-1 infection (47, 48), and decreased in inflammatory responses. The LXR/RXR pathways have been shown to inhibit HIV infection (49) and inflammatory responses (50). This is in contrast with the 6h timepoint when pathways associated with dendritic cell maturation and NFκB signaling were up regulated, both suggesting an increase in inflammation in the tissue.

### Upstream Regulator Analysis Shows the Dynamics of the Cervical Mucosal Transcriptome After HIV Exposure With a Transient Expression of Many Genes That Is Lost at 24h

**Figure 4:**
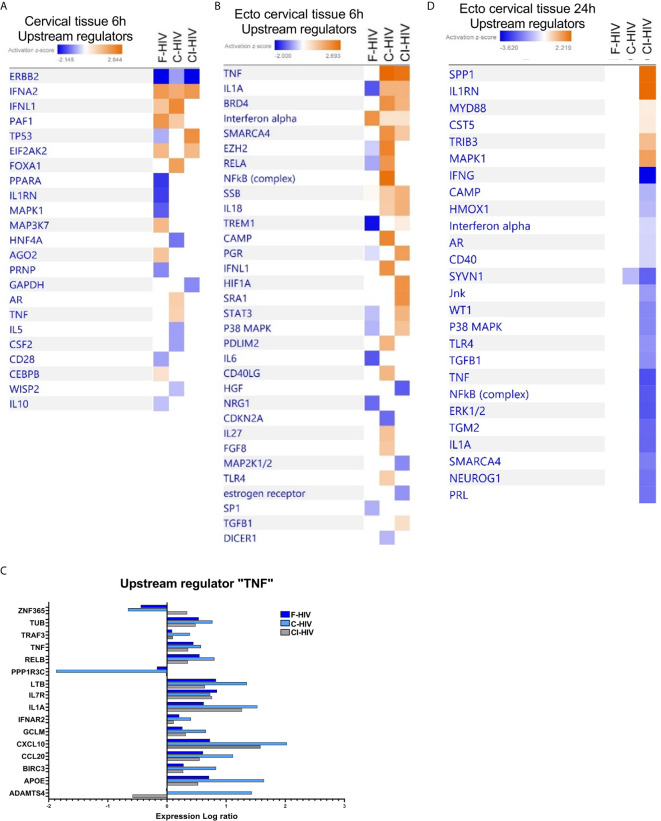
Upstream regulator analysis showing activation and upregulation of several genes induced by HIV in the cervical mucosal tissue 6h post exposure that is lost at 24h. Cervical mucosal biopsies (3mm) were spin-occulated with 250ng/ml F-HIV, C-HIV; CI-HIV or mock for 2 hours. The biopsies were then washed and incubated for an additional 4h or 22h. The tissues were harvested, and RNA was purified and whole transcriptome sequencing was performed. **(A)** Analysis of upstream regulators affected by the HIV exposure in the complete, i.e. endo and ecto, cervical tissue at 6h were assessed by IPA and presented as a heat map with the threshold for p-values set to log_2_1.3 presented as an activation Z-score with a cutoff of 0.5 Z-score. **(B)** Analysis of upstream regulators affected by the HIV exposure in ectocervical tissue at 6h were assessed by IPA and presented as a heat map with the threshold for p-values set to log 1.3 presented as activation Z-score with a cutoff of 0.2 Z-score. **(C)** Analysis of upstream regulators affected by the HIV exposure in ectocervical tissue at 24h were assessed by IPA and presented as a heat map with the threshold for p-values set to log 1.3 presented as activation Z-score with a cutoff of 0.2 Z-score. **(D)** Analysis of expression log ratio of factors grouped as regulators of TNF from figure 4b. (N=6).

The transcriptome profile of upstream regulators in the cervical tissue, the combined ecto and endocervix, 6h after HIV-1 exposure clearly displayed that the different forms of HIV shaped distinct transcriptomic profiles. F-HIV showed significant positive activation Z-scores for the upstream regulators IFNA2, MAP3K7 and CEBPB. In the opsonized HIV groups, significant positive activation Z-scores for IFNL1, FOXA1, AR, and TNF were found in C-HIV, whereas activation of TP53 and EIF2AK2 were predicted by exposure to CI-HIV **(** **Figure 4A** **)**. In ectocervix, the impact of TNF in response to C-HIV exposure was even stronger than seen for the combined ecto and endocervix, which was reflected in that most molecules assigned by IPA to the TNF upstream regulator pathway had higher fold change in tissues exposed to C-HIV than in tissues exposed to F-HIV and CI-HIV **(** **Figures 4B, C** **)**. The C-HIV exposed ectocervical tissue also had positive Z-scores for e.g., BRD4, cAMP and NF-κB (complex) and significant P-values. There was a bigger overlap between CI-HIV and C-HIV exposed tissues than with F-HIV **(** **Figure 4B** **)**. The opsonized HIV groups and shared the profile for TNF, BRD4, IL1A, SMARCA4, SSB and IL18. In addition, C-HIV also induced activation of PGR, HIF1A, SRA1, and TGFB1 **(** **Figure 4B** **)**. IL-18 can induce TNF production (51) and TNF conditioning reportedly increases the expression of CCR5 on DCs (13, 52). At 6h post HIV exposure, our data suggest that complement-opsonized viruses induced a more potent activation of immunological signaling in the cervical tissue, compared to the F-HIV. At 24h post HIV exposure the transcriptomic profiles had changed. The effects on the tissues induced by the F-HIV and C-HIV exposure were abolished and the profiles of the CI-HIV exposure had changed **(** **Figure 4D** **)**. CI-HIV divulged positive Z-scores for SPP1, IL1RN, MYD88, CST5, TRIB3 and MAPK1 signaling suggesting that the antibodies added during complement opsonization also affect gene expression in response to HIV exposure. Negative Z-scores of upstream regulators such as cAMP, IFNγ, TNF and IFNα post CI-HIV exposure, and no change for F-HIV and C-HIV, suggest that all forms of HIV either suppress signaling or lay silently in the ecto cervical tissue at the 24h time-point **(** **Figure 4D** **)**. In summary, upstream regulator analysis show that F-HIV, C-HIV and CI-HIV rapidly induce distinct gene expression patterns that are significantly changed within 24 hours.

### Complement-Opsonized HIV Activates Infection and Viral Replication Pathways/Groups in the Ectocervical Tissue

**Figure 5:**
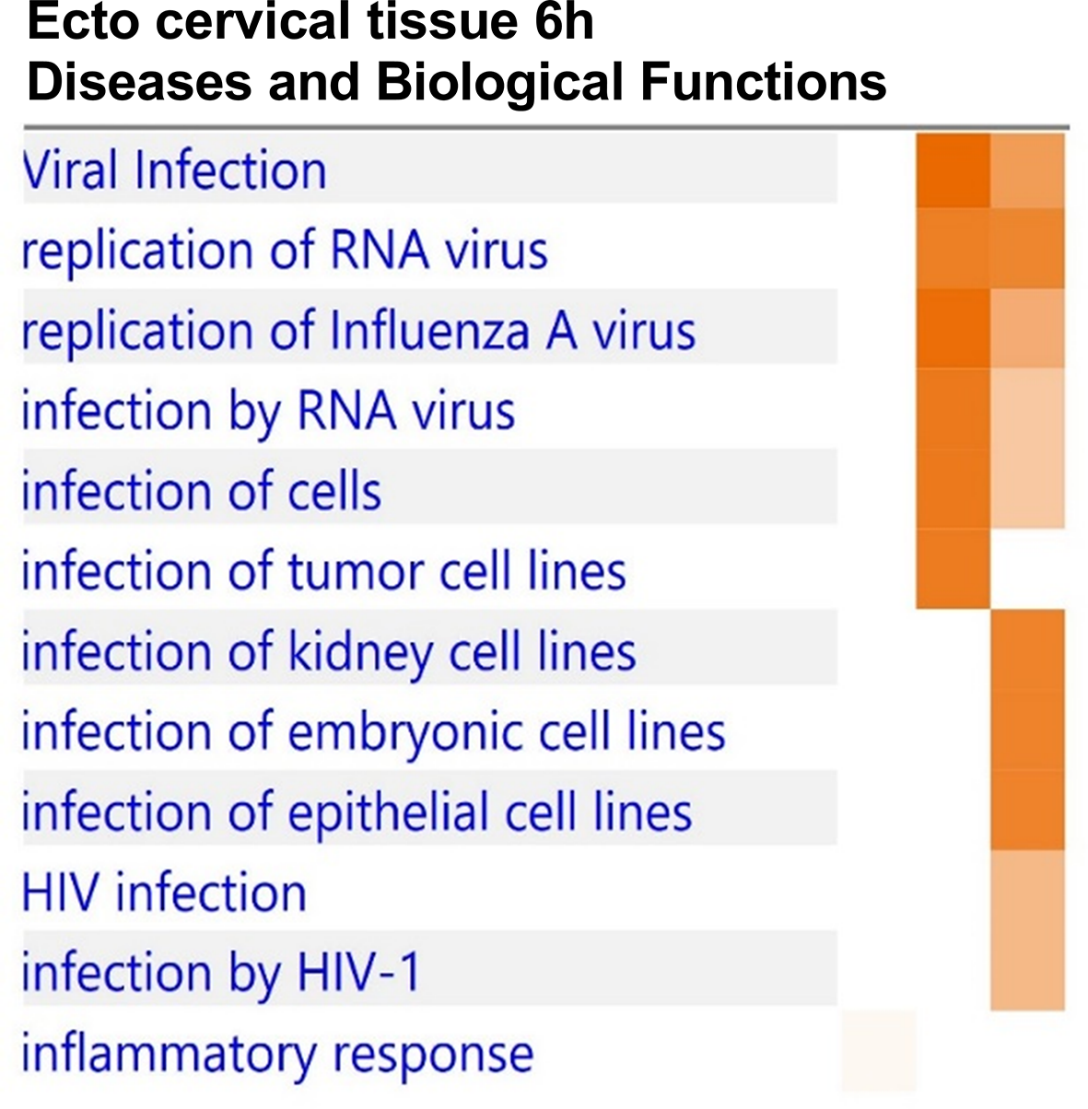
Complement-opsonized HIV activates infection and viral replication pathways/groups in the ectocervical tissue. Cervical mucosal biopsies (3mm) were spin-occulated with 250ng/ml F-HIV, C-HIV; CI-HIV or mock for 2 hours. The biopsies were then washed and incubated for an additional 4h. The tissues were harvested, and RNA was purified and whole transcriptome sequencing was performed. Analysis of the RNA seq transcriptome was analyzed in the IPA using the Biofunctions and Diseases analysis for enrichment of groups/pathways involved in infection and viral replication and presented as a heat map with the threshold for p-values set to log 1.3 (p < 0.05) presented as activation Z-score. (N=6).

The RNA seq transcriptome data of the ectocervical tissue exposed to HIV-1 for 6h was analyzed in the IPA function “Diseases and Biological Functions” for enrichment of groups/pathways involved in infection and viral replication. We found positive Z-scores for several infection and viral replication pathways/groups after exposure to complement-opsonized HIV-1 but not after F-HIV exposure **(** **Figure 5** **)**, clearly demonstrating that opsonization altered HIV’s effects on the cervical mucosa gene expression. The C-HIV had a higher Z-score for “viral infection”, “infection of cells”, “infection by RNA viruses” and “replication of influenza virus” **(** **Figure 5** **)**. In addition, the pathways called “infection by HIV-1” and “HIV infection” showed positive Z-scores for the CI-HIV group **(** **Figure 5** **)**.

### Free and Complement- Opsonized HIV Exposure Have Diverse Effects on Expression of Proteins Involved in Canonical Pathways

**Figure 6:**
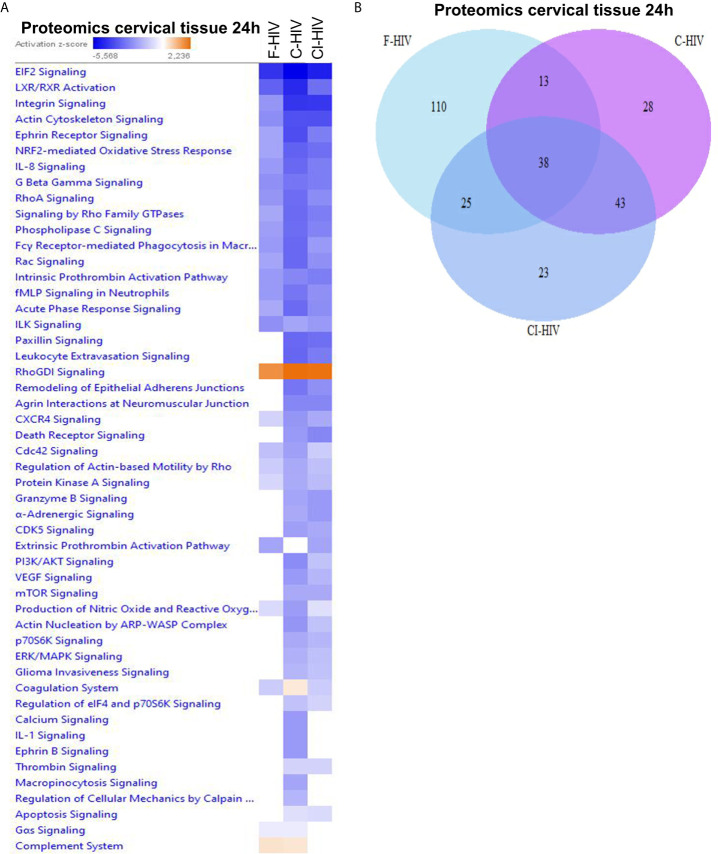
Free and complement-opsonized HIV exposure have diverse effects on expression of proteins involved in canonical pathways. Ecto-cervical biopsies were treated with F-HIV, C-HIV, CI-HIV, or mock treated for 24h. The samples were lysed and digested into peptides that was labeled with iTRAQ isobaric and analyzed using LC/MS. The resulting data were then normalized to the mock samples and **(A)** canonical pathways affected were analyzed by IPA and presented as a heat map with the threshold for p-values set to log 1.3 (p < 0.05) and presented as activation Z-score. **(B)** Venn diagram analysis was performed for the canonical pathways activated by F-HIV, C-HIV, CI-HIV to identify similarities in pathway activation. (N=5).

Ectocervical biopsies were exposed to mock, F-HIV, C-HIV or CI-HIV and cultured for 24h after which they were lysed, and LS-MS proteomics was performed. The proteomics was only performed at 24h, due to that we expected low effects on protein at the 6h timepoint. We identified 2086 proteins in total, and data were normalized by subtracting each sample’s reference (mock). There were 1,302 proteins consistently measured across all samples, and these were compared between viral treatment groups. There were 40 proteins (3.1%) that were differentially abundant between the different viral groups (p<0.05, Friedman test) (**Table 1**). However, there were no proteins that passed multiple hypothesis testing correction. All proteomic data were thereafter analyzed using the IPA software to assess each viral treatment’s effects on protein pathways and functions. Visualization of treatment specific protein expression was done by heat maps with Z-scores **(** **Figure 6A** **)**. Most Canonical pathways were inhibited in the HIV treated groups with the exception for pathways such as “RhoGDI signaling”, which had a positive Z-score for all with the highest for C-HIV and CI-HIV and complement system, which had a positive Z-score for F-HIV and C-HIV **(** **Figure 6A** **)**. Of the top canonical pathways activated after F-HIV treatment, the most affected pathways were EIF2 Signaling, and pathways involved in T-cell associated signaling such as Phospholipase C Signaling and hormonal signaling such as Ephrin Receptor Signaling, all with negative Z-scores suggesting inhibition **(** **Figure 6A** and **Table 2** **).** C-HIV on the other hand, gave negative Z-scores for several pathways associated with endocytosis such as “Actin Cytoskeleton Signaling” and “Integrin Signaling” and metabolomic signaling, including “RXR related pathways”. C-HIV also affected the remodeling of epithelial adherence junctions **(** **Figure 6A** and **Table 3** **)**. CI-HIV affected EIF2 signaling and metabolic pathways where RXR and PKA signaling was some of the most affected pathways with negative Z-score **(** **Figure 6A** and **Table 4** **)**. Comparing the different HIV groups showed distinct patterns with some degree of overlap **(** **Figure 6B** **)**. Accordingly, proteomic analysis of cervical tissue exposed to different forms of HIV showed a general inhibition of canonical signaling pathways for all viral treatments. To establish if this profile was preserved even later in the HIV-1 exposed tissue we analyzed the released proteins in supernatants using the proteomics panels Inflammation and Immune Response at 96h. The protein profiles at 96h showed a decrease of many proteins and thereby verifying the GS-MS proteomics (**Supplementary Figure 3** **).**

**Table 1:** Proteins differentially abundant between viral treatment arms.

Protein Name	Accession ID	Average FHIV	Average CHIV	Average CIHIV	Friedman p-value	General Function
**Calpain-2 catalytic subunit**	CAN2_HUMAN	-0,031	0,092	-0175	0,0001	Regulation of cytoskeleton organization
**Flotillin-2**	FLOT2_HUMAN	-0,191	0,221	0,033	0,006	Epidermal cell adhesion
**Poly [ADP-ribose] polymerase 1**	PARP1_HUMAN	-0,074	0,148	0,091	0,007	Transcription regulation
**Cytokine c**	CYC_HUMAN	-0,324	-0,323	0,022	0,008	Cellular
**Signal transducing adapter molecule 2**	STAM2_HUMAN	-0,078	0,456	-0,017	0,01	Protein transport
**Cytosolic non-specific dipeptidase**	CNDP2_HUMAN	-0,165	0,045	-0,186	0,01	Proteolysis
**RNA-binding motif protein, X**	RBMX_HUMAN	-0,102	0,111	0,075	0,013	mRNA processing
**Thiosulfate sulfurtransferase**	THTR_HUMAN	-0,072	0,433	-0,066	0,014	rRNA transport
**Erlin-2**	ERLN2_HUMAN	-0,004	0,203	-0,04	0,014	Lipid metabolism
**Histone H2A,V**	H2AV_HUMAN	-0,288	0,165	0,042	0,016	Chromatin organization
**Ras-related protein Rab-35**	RAB35_HUMAN	0,042	0,516	0,095	0,017	Antigen processing and
**Matrix-remodeling-associated ptotein 7**	MXRA7_HUMAN	0,12	0,674	0,199	0,021	Unknown
**Talin-2**	TLN2_HUMAN	0,015	0,452	0,357	0,021	Cell adhesion
**Splicing factor, proline-and**	SFPQ_HUMAN	0,015	0,165	0,075	0,021	Innate immunity
**Catechol O-methyltransferase**	COMT_HUMAN	-0,01	0,247	0,053	0,021	Catecholamine metabolism
**Band 4,1-like protein**	E41L2_HUMAN	-0,048	0,226	-0,009	0,021	Cell cycle
**Nucleolar protein 58**	NOP58_HUMAN	0,189	0,42	0,02	0,021	rRNA processing
**Basigin**	BASI_HUMAN	-0,247	0,102	-0,23	0,021	ECM disassembly
**Histone H1,10**	H1X_HUMAN	-0,131	0,443	0,329	0,026	Chromosome condensation
**Ras-related protein Rab-58**	RAB5B_HUMAN	-0,097	0,43	-0,065	0,027	Antigen processing and
**Myosin-9**	MYH9_HUMAN	-0,223	-0,078	0,046	0,027	Cell adhesion
**Protein NDRG1**	NDRG1_HUMAN	-0,074	0,171	-0,046	0,029	Mast cell
**Membrane-associated progesterone**	PGRC1_HUMAN	0,118	0,716	0,385	0,029	Neutrophil degranulation
**Vacuolar protein sorting -associated**	VP26A_HUMAN	-0,21	0,182	0,13	0,029	Protein transport
**Reticulon-4**	RTN4_HUMAN	-0,145	0,409	0,171	0,03	Neurogenesis
**Protein LYRIC**	LYRIC_HUMAN	-0,052	0,375	0,084	0,033	Positive regulation of NF-kappa B transcription
**Inactive tyrosine-protein kinase 7**	PTK_HUMAN	0,068	0,301	0,194	0,033	Cell adhesion
**Rootletin**	CROCC_HUMAN	0,13	0,485	0,126	0,035	Epithelial structure
**Nuclear factor 1 C-type**	NFIC_HUMAN	-0,199	0,228	0,083	0,035	Transcription regulation
**Spermine synthase**	SPSY_HUMAN	-0,164	0,187	0,126	0,039	Polyamine biosynthesis
**Keratin, type II cytoskeletal 78**	K2C78_HUMAN	0,053	-0,107	-0,449	0,039	Keratinization
**Suprabasin**	SBSN_HUMAN	-0,048	0,295	-0,7	0,04	
**Aldehyde dehydrogenase family 1 member A3**	AL1A3_HUMAN	0,001	-0,298	-0,019	0,041	Retinoic acid metabolism
**E3 SUMO-protein ligase RanBP2**	RBP2_HUMAN	-0,073	0,222	-0,045	0,042	mRNA transport
**Phenylalanine--tRNA ligase alpha subunit**	SYFA_HUMAN	-0,042	0,159	0,077	0,043	Potein biosynthesis
**Calpain small subunit**	CPNS1_HUMAN	-0,182	-0,194	-0,504	0,045	ECM disassembly
**Extended synaptotagmin-1**	ESYT1_HUMAN	-0,295	0,072	-0,082	0,046	Lipid transport
**Nidogen-1**	NID1_HUMAN	0,002	-0,006	0,234	0,047	Cell adhesion
**Pinin**	PININ_HUMAN	0,034	0,129	-0,057	0,049	Cell adhesion
**60S ribosomal protein L10a**	RL10A_HUMAN	-0,162	0,063	0,057	0,05	Viral transcription

**Table 2:** Biofunctions in F-HIV treated cervical mucosa tissue.

Diseases or Functions Annotation	p-Value	Activation z-score	Molecules	# Molecules
**Proliferation of connective tissue cells**	4.19E-02	-0.849	CAPNS1,CDH13,DNM1L,GRB2,PRDX4	5
**Immune response of cells**	3.28E-02	0.200	DNM1L,GRB2,IGHA1,IGHA2,SERPINB9	5
**Cell death of connective tissue cells**	4.15E-02	0.305	CAPNS1,COX5A,DNM1L,GRB2,UBE2K	5
**Viral Infection**	1.84E-02	0.726	AFG3L2,DNM1L,FGA,GRB2,GYG1,IGJ,MTX1,NHP2L1, RBM17,SERPINB9,SNRPA1	11
**Cell death of tumor cell lines**	2.74E-02	0.741	CAPNS1,CCT5,CD99,COX5A,DNM1L,LGALS3BP,PRKA R1A,RBM17,RPLP0,UBE2K	10
**Necrosis**	2.31E-02	1.087	ALDH2,CAPNS1,CAPRIN1,CCT5,CD99,COX5A,DNM1L, FGA,GRB2,LGALS3BP,PRKAR1A,RBM17,RPLP0,SERPI NB9,UBE2K	15
**Cell death**	6.21E-03	1.378	AFG3L2,ALDH2,AP1G1,CAPNS1,CAPRIN1,CCT5,CD99, COX5A,DDX19A,DNM1L,FGA,GRB2,LGALS3BP,PRDX4, PRKAR1A,RBM17,RPLP0,SCRIB,SERPINB9,UBE2K	20

Proteomics data from cervical biopsies treated with F-HIV were normalized to the mock value for each donor and analyzed using. Ingenuity IPA (Qiagen) to determine the top biofunctions associated with this treatment.

**Table 3:** Biofunctions in C-HIV treated cervical mucosa tissue.

Diseases or Functions Annotation	p-value	Activationz-score	Molecules	# Molecules
**Cell cycle progression**	1.07E-02	-1.964	APOE,CKAP5,CUL2,DIABLO,FASN,MECP2,NUMA1,PRK AR2B,PTMA,SCRIB	10
**Cell death of melanoma cell lines**	1.19E-02	-0.594	CUL2,DIABLO,RAB35,YBX1	4
**Apoptosis**	1.41E-02	-0.366	APOE,CASP14,CKAP5,CUL2,DDX19A,DIABLO,FASN,H2AFX,LAMA2,MECP2,NUMA1,PDXK,PPP3CA,PRKAR2B,PTMA,SCRIB,SUN1,YARS,YBX1	19
**Cell death of tumor cell lines**	9.67E-03	-0.222	APOE,CASP14,CCT5,CKAP5,CUL2,DIABLO,FASN,H2AFX,PRKAR2B,PTMA,RAB35,YARS,YBX1	13
**Proteolysis**	1.57E-03	-0.152	APOE,ASPH,CASP14,PITRM1,PREP,SPINK5	6
**Catabolism of protein**	2.45E-03	-0.152	APOE,ASPHE,CASP14,CUL2,HGS,PITRM1,PREP,SPINK5	8
**Metabolism of protein**	1.80E-03	0.640	APOE,ASPH,CASP14,CKAP5,CUL2,HGS,PITRM1,PREP,SPINK5,UPF3B,YBX1	11
**Endocytosis**	2.71E-04	1.067	ANKFY1,APOE,ATP6VOD1,HGS,PPP3CA,RAB34,SCRIB	7
**Proliferation of cells**	3.07E-04	1.125	APOE,ASPH,ASPSCR1,ATP6VOD1,CCT5,CES2,CUL2,DIABLO,FASN,H2AFX,HGS,LAMA2,MECP2,NCCRP1,NOP58,NUMA1,OSTF1,PDAP1,PDXK,PPP3CA,PREP,PRKAR2B,PTMA,RAB35,SCRIB,SF1,SRSF5,YARS,YBX1	29
**Proliferation of fibroblast cell** **lines**	2.40E-02	1.446	ASPSCR1,CES2,NOP58,PRKAR2B,PTMA	5
**Proliferation of prostate cancer** **cell lines**	1.55E-02	1.951	FASN,HGS,PRKAR2B,YBX1	4
**Size of body**	2.64E-02	1.974	APOE,DIABLO,H2AFX,LAMA2,MECP2,PPP3CA,PRKAR2B,SUN1	8
**Organization of cytoplasm**	2.26E-02	2.597	ANXA8/ANXA8L1,APOE,CKAP5,CROCC,FASN,LAMA2,MECP2,NSFL1C,NUMA1,PTK7,RAB35,YBX1	12

Proteomics data from cervical biopsies treated with F-HIV were normalized to the mock value for each donor and analysed using. Ingenuity IPA (Qiagen) to determine the top biofunctions associated with this treatment.

**Table 4:** Biofunctions in CI-HIV treated cervical mucosa tissue.

Diseases or Functions Annotation	p-value	Activation z-score	Molecules	#Molecules
Perinatal death	3.29E-02	-1.387	CAPRIN1,CTNNA2,RAP1B,TOP2B,YBX1 CAPRIN1,COL5A1,CTNNA2,FOSL2,GPX3,HISTlH1C,M	5
Organismal death	2.13E-03	-1.201	TAP,MUC5B,PRKACA,PTGES,RAP1B,RPL24,SF3B1,SRR T,TOP2B,YBX1	16
Neonatal death	3.92E-02	-1.067	CAPRIN1,CTNNA2,TOP2B,YBX1	4
Proliferation of tumor celllines	3.39E-02	-0.432	CES2,CLIP11,GKC,MTAP,PRKACA,PTGES,RAP1B,RPS14 ,YBX1	9
Edema	1.53E-02	-0.277	DBNL,PRKACA,PTGES,YBX1	4
Growth Failure	3.42E-02	-0.277	FOSL2,HIST1H1C,MUC5B,PRKACA,YBX1 ADD3,AG01,CAPRIN1,CASP14,CCT5,CTNNA2,CUL2,F	5
Cell death	5.14E-03	-0.255	OSL2,HIST1H1C,IGKC,MUCSB,NAE1,PDCD5,PRKACA, PTGES,RAP1B,SRRT,TOP2B,YBX1	19
Microtubule dynamics	2.54E-03	-0.176	CAPRIN1,CDC42BPB,CLIP1,CTNNA2,DBNL,FLOTl,PRK ACA,RAP1B,TOP2B,YBX1	10
Apoptosis	3.14E-02	-0.067	AG01,CAPRIN1,CASP14,CUL2,HIST1H1C,MUC5B,NAE 1,PDCD5,PRKACA,PTGES,RAP1B,SRRT,TOP2B,YBX1	14
Expression of RNA	2.97E-02	-0.014	AG01,ALYREF,CAPRIN1,FOSL2,GCDH,HIST1H1C,NAE1 ,PRKACA,RPL24,RPS14,SRRT,YBX1	12
Proliferation of cells	2.48E-03	0.672	AG01,CAPRIN1,CCT5,CDC42BPB,CES2,CLIP1,CMA1,C UL2,FLOTl,FOSL2,GPX3,1GKC,MTAP,NAE1,PRKACA,P TGES,RAP1B,RPS14,SRRT,TOP2B,YBX1	21
Replication of Influenza A virus	8.44E-03	1.131	CDC42BPB,PRKACA,RPS14,SF3B1	4
HIV infection	1.23E-03	1.212	COL5A1,MUC5B,RAP1B,SF3B1,SRRT,TM9SF2T, OP2B, YBX1	8
Infection by HIV-1	2.35E-03	1.212	COL5A1,MUC5B,RAP1B,SF3B1,SRRT,TM9SF2,YBX1	7
Infection of cells	1.07E-02	1.212	COL5A1,MUC5B,RAP1B,SF3B1,SRRT,TM9SF2,YBX1	7
Size of body	4.69E-02	1.706	DBNL,HIST1H1C,PRKACA,PTGES,RAP1B,TOP2B	6
Replication of RNA virus	6.20E-04	1.964	AG01,CDC42BPB,IGKC,PRKACA,RPS14,SF3B1,YBX1	7
Proliferation of fibroblasts	1.58E-02	1.969	FOSL2,PTGES,RAP1B,YBX1	4
Infection of embryonic cell lines	6.71E-03	2.000	COL5A1,SF3B1,SRRT,YBX1	4
Infection of epithelial cell lines	6.71E-03	2.000	COL5A1,SF3B1,SRRT,YBX1	4
Infection of kidney cell lines	7.62E-03	2.000	COL5A1,SF3B1,SRRT,YBX1	4
			AG01,CDC42BPB,COL5A1,HIST1H1C,IGKC,MTX1,MU	
Viral Infection	1.84E-05	2.094	C5B,PRKACA,RAP1B,RPS13,RPS14,SF3B1,SRRT,TM9S F2 TOP2B YBX1	16

Proteomics data from cervical biopsies treated with F-HIV were normalized to the mock value for each donor and analyzed using Ingenuity IPA (Qiagen) to determine the top biofunctions associated with this treatment.

### Proteins Associated With Viral Infection Are Upregulated in Cervical Mucosa Exposed to Complement-Opsonized HIV

**Figure 7:**
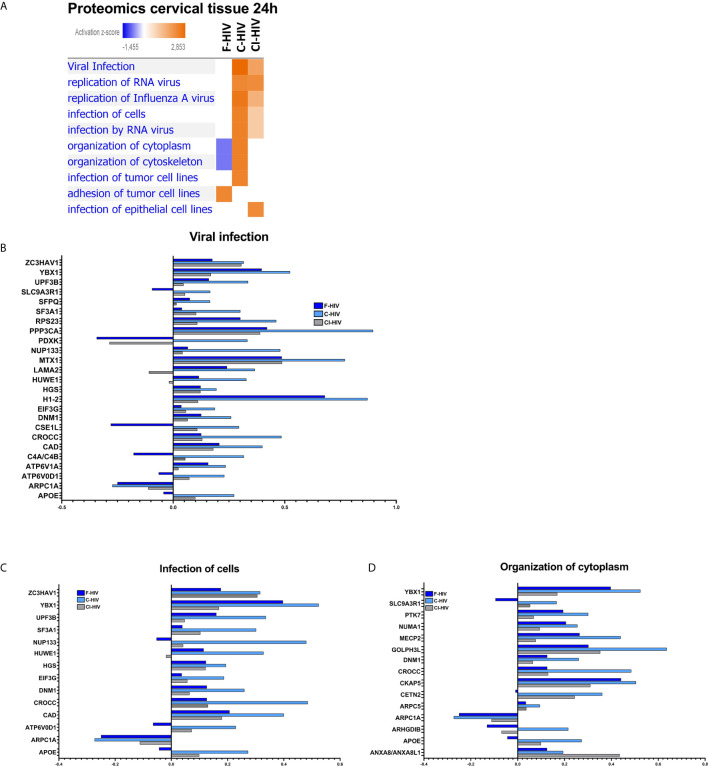
Proteins associated with viral infection is upregulated in cervical mucosa exposed to complement-opsonized HIV. Ectocervical biopsies treated with F-HIV, C-HIV, CI-HIV, or mock treated for 24h.The samples were lysed and digested into peptides that was labeled with iTRAQ isobaric and run on LC/MS. The resulting data were then normalized to the mock samples. **(A)** Affected proteins was thereafter analyzed in the IPA function “Diseases and Biological Functions” for visualization of enrichment of groups/pathways involved in infection and viral replication and presented as a heat map with the threshold for p-values set to log 1.3 (p<0.05) presented as activation Z-score. **(B)** Analysis of protein log ratio of factors involved in the “Diseases” pathway viral infection from figure 7A. **(C)** Analysis of protein log ratio of factors involved in the “Diseases” pathway Infection of cells from figure 7A. **(D)** Analysis of protein log ratio of factors involved in the “Diseases” pathway Organization of cytoplasmic molecules from figure 7A. (N= 6).

When investigating “Diseases and Biological functions” in IPA, it was a clear distinction in the activation of pathways related to viral biofunctions between the complement-opsonized virus and F-HIV **(** **Figure 7** **)**. Both the complement-opsonized forms of HIV activated pathways involved in viral infection, replication of and infection by RNA viruses to a much higher degree than F-HIV. In addition, complement treatment also induced pathways involved in the organization of cytoplasm and cytoskeleton **(** **Figure 7A** **)**. An in-depth investigation of factors involved in different pathways in “Disease and Biological Function” was performed **(** **Figures 7B–D** **).** A clear pattern, showing a higher expression log ratio for C-HIV and CI-HIV for almost all molecules assigned by IPA into the pathways “Viral infection”, and “Infection of cells” **(** **Figures 7B, C** **)** and for several others involved in viral infection and replication, with a high overlap in molecules between C- and CI-HIV was observed (data not shown). In addition, the pathway “Organization of cytoplasm” and “Organization of cytoskeleton” showed highest expression log ratio for C-HIV **(** **Figure 7D** and data not shown). In conclusion the data clearly states an increase in viral infection of tissues exposed to C- and CI-HIV compared to F-HIV, most likely as a result of the different signaling cascades induced in the tissues by the initial exposure to the different viral compositions.

## Discussion

The cervical mucosa represents the most common route for HIV transmission and most of all new infections occur in women (1, 2). The effects of initial HIV exposure, i.e., the first 24h, on the cervical tissue has not been well elucidated and was therefore the scope of the current investigations. By using RNA sequencing and LS-MS proteomics to get the transcriptome and proteomic profiles, respectively, the overall effects HIV exposure exerted on the cervical mucosal tissue were investigated. Considering the stringent statistical analyses adopted, the findings assume paramount importance for advancing our understanding of the events during the initial phases of exposure to HIV and the establishment of infection. To start with, we verified that the complement-opsonized HIV presented a higher level of infection of the cervical tissue compared to free HIV and in the emigrating cells had a preference to infect DCs over CD4 T cells, which is in agreement with previous finding from our laboratory (21). The higher HIV-1 infection of DCs is in line with findings from our and several groups for monocyte derived DCs (53–56). The observed increased HIV infection of the emigrating cells with the complement-opsonized virus, was associated with a significant upregulation of the pathways and factors involved in the diverse aspects of viral infection, viral entry, and viral replication, both at the transcriptome as well as the proteome levels in the cervical mucosa and should reflect the effects *in vivo* as the transferred HIV virions should be complement-opsonized (17). Importantly, the effects seen in the tissues on the transcriptome and protein levels are not restricted to a direct infection of immune cells, but could also be attributed to the activation of PRRs and other innate pathways in the epithelial cells and immune cells that are exposed to but not productively infected by HIV but produce pro-inflammatory chemokines and anti-viral factors as shown by other groups (4, 57, 58). Thus, the effects seen in the tissues on the transcriptome and protein levels in this study are likely to be attributed to the activation of mucosal cells and not restricted to a direct infection of immune cells. Besides the activation of PRRs the virions interactions with target cell in the mucosa involve an array of receptors including a4b7 for CD4 T cell infection (59) and Siglec-1 for DCs capture and transfer of cell associated virus to new cells (60). This important early role of DCs and CD14+ myeloid cells in the capture the HIV and harboring of infectious virions and in the transmission of HIV to CD4+ T cells (29) might also play an important role in our system. Besides the role of DCs in capturing the virions in the upper epithelium, Rhodes et al. showed that HIV-1 penetrated to the sub-epithelial layer where the myeloid cells, i.e., CD14+ CD1c+ monocyte derived DCs and cDC2s, became HIV-1 infected and transmitted the virus to CD4+ T cells (30). Integrated HIV DNA was found early in T cells but not myeloid cells indicating the initial replication occurred in the T cells not myeloid cells in the cervical mucosa (29). We found that both the mucosal T cells and DCs to productively infected after three days and this is accordance to findings by several other groups (1, 2, 28, 31, 37, 38).

There are differences in tissue composition throughout the female genital tract, i.e. between the endocervix and ectocervix, and it is clear that both these sites are targets for HIV infection (61). We found infection in the cervical endo and ecto tissue and in emigrating DCs and T cells, with higher infection induced by complement-opsonized HIV in the tissue and DCs compared to infections with free virions, which confirms our previous findings (21). The immune response of cervical mucosa is affected by the menstrual cycle (62). A “window of vulnerability” to HIV-infection during the secretory phase has been proposed (63). The majority of tissue donors in this study were older than 45 years, i.e., pre-menopausal or menopausal. Thus, we consider the menstrual cycle to have little or no impact on HIV infectivity in our model.

Proinflammatory cytokines and chemokines are constitutively expressed in the female genital tract (64, 65), which has been considered to be immunologically active (65). This may explain why only restricted changes of many inflammatory factors in response to HIV were observed at the protein level, despite the triggering of inflammatory responses at the on gene/mRNA level. Another important aspect of the initial events on both mRNA and protein levels, during the early phases of HIV exposure, is the responses exerted by the epithelial cells lining the cervical mucosa. SIV transmission *via* vaginal mucosal challenge has been shown to induce MIP-3α/CCL20 signaling in endocervix in combination with innate immune and inflammatory responses to infection in both cervix and vagina (66). In the initial response after SIV exposure, the cervical mucosal epithelium played an important role in the production of inflammatory chemokines including CCL3, CCL20 and CXCL8 (58). These chemokines are crucial for the recruitment of macrophages, plasmacytoid DCs and CD4+ T cells to the site of infection, which preferentially are clustered below the cervical epithelium in a chemokine dependent manner (58). In addition, SIV rapidly induced a broad spectrum of proinflammatory responses in the epithelium of the cervical mucosa in rhesus macaques, characterized by an upregulation of the NF-kB signaling pathway and a reduction in the glucocorticoid receptor signaling pathway mainly in the epithelial cells lining the mucosa (67). In our data we also found an upregulation of CCL20, CCL2 and CXCL11 after 6h of HIV exposure. In addition, we could measure an increase in CXCL8 on mRNA level induced by F-HIV, and on protein levels for all HIV groups. At the 24h time point, transcription of these chemokines was downregulated. Thus, it can be hypothesized that the expression of cytokines seen in the SIV model might be driven by immune cells migrating into the tissue. In accordance with Shang et al. (67) NF-kB signaling was upregulated during the early phase of HIV exposure and establishment of infection. Combined, these results suggest that female genital tract epithelial cells are of major importance in our system since their modulation of the local mucosal environment will affect the recruitment of immune cells and the establishment of HIV infection.

HIV exposure has previously been shown to induce a transient activation of immune responses in different models (22, 46, 68), which is in line with our present findings. The initial events and activation of e.g., inflammatory responses in the mucosal tissue at the 6h post HIV exposure may facilitate the establishment of infection. It has been reported that inflammation, even at low asymptomatic levels, could increase the risk of HIV and SIV acquisition (66, 69, 70). Transcriptomics showed an activation of inflammatory pathways in the tissues exposed to complement-opsonized HIV at 6h. Enrichment of factors related to inflammatory pathways were not reflected at the protein levels at 24h. Active suppression of mRNA translation into protein caused by, for instance micro RNAs may contribute to lack of congruence (71), as well as proteasomal protein degradation *via* ubiquitination, a pathway know to be exploited by HIV (72), or a threshold value of mRNA levels for protein synthesis that is not reached (73). HIV has previously been shown to modulate mRNA processing and translation at multiple levels and to redirect protein synthesis toward a more beneficial state for viral replication and dissemination (68).

In the combined endo and ecto cervical tissues, pathway analysis showed the activation of the IFN signaling pathway with IFNA and IFNL1 as top enriched upstream regulators after 6h exposure. The enrichment of factors related to IFNA and IFNL1 was more pronounced in tissues exposed to F-HIV than C-/CI-HIV at this time point. This is in accordance with previous findings, wherein we have shown a CR3-mediated suppression of antiviral pathways in DCs exposed to complement-opsonized HIV (22).

Metabolic changes have been studied in many aspects of inflammation and infection, but not in cervical tissues in the context of HIV infection. After HIV exposure, there was enrichment of factors of the PPAR and LXR/RXR pathways at the 24h time-point. Both transcriptomics and proteomics indicated activation of the LXR/RXR signaling pathways. Resting and memory immune cells are mainly metabolically inactive but can after stimulation rearrange their metabolism towards a faster production of ATP and important building blocks for the induction of a strong immune response (74). LXRs forms heterodimers with RXRs and the complex acts as a modulator of lipid and cholesterol homeostasis as well as inflammatory signaling (75). LXR signaling is associated with repression of inflammation through inhibiting NF-κB (50). Activation of these pathways are therefore indicative of active suppression of inflammatory signaling in tissues exposed to HIV. The transcriptomic data also suggest inhibition of inflammation after 24h of HIV exposure, since there were negative Z-scores, i.e., inhibition/suppression for “Toll-like Receptor signaling” and “Role of Pathogen Recognition Receptors in Recognition of Bacteria and Viruses” at this timepoint. The anti-inflammatory signaling at 24h was most pronounced after exposure to complement-opsonized HIV. The protein kinase A (PKA) signaling pathway (47, 76, 77), was activated for C-HIV and CI-HIV. The PKA together with cAMP signaling is an important modulator of many different pathways involved in immune activation and inhibit transcription of several inflammatory cytokines induced by the NF-κB pathway (77). Furthermore, cAMP have previously been shown to be increased in HIV positive individuals (76, 77). Together these data suggest suppression of the cervical tissue inflammatory response, and possibly a shift to an immunosuppressive milieu in the tissue, preferentially induced by opsonized HIV at 24h post exposure.

HIV opsonized by serum and serum-containing HIV-specific antibodies increase the internalization of virions *via* receptor-mediated endocytosis (56, 78) and the receptors involved in the viral interaction and uptake. The receptors involved in endocytosis include complement receptors and Fc-receptors. Increased levels of proteins involved in endocytosis after C-HIV exposure, in the cervical tissue explants, may correlate with increased viral uptake and increased viral dissemination. We have previously shown that C-HIV binding and signaling *via* complement receptor 3, induce suppression of the antiviral and the inflammatory response in immature DCs *via* the complement receptor 3 engagement (22). This was mainly due to fixation of iC3b molecules on the viral surface during HIV opsonization (19). The effects complement HIV-1 exert on the DCs has been shown to depend on the balance of CR3 and CR4 on the DCs, with high level of CR3 give rise to suppression of inflammation and antiviral activities, whereas high CR4 induce and activation (79). The presence of pre-existing HIV specific antibodies such as anti-gp120 and anti-gp41 during complement opsonization will increase C1q deposition on the viral surface (20). Activation of Fc-receptors by the complement-opsonized virions will also influence the ensuing response (80). For example, an activation of FcγRIIB induced by the formation of SIV specific immune complexes following SIV-vaccination of rhesus macaques have been shown to induce immune suppression in the mucosal epithelium (67). Modulation of Fc-receptor signaling might also have affected the present findings, since these receptors can transduce a wide variety of different immunological and HIV specific effector functions (80). The differences in pathways activated by C-HIV and CI-HIV could be attributed to differences in Fc-receptor engagement. In addition, differences in micro-RNA expression between C-HIV and CI-HIV exposed tissues may contribute to differences in inflammatory signaling by modulation of Fc-receptor induced signaling.

In addition to receptor-mediated immune suppression, there may also be a regulation of translation of mRNA into protein *via* miRNAs. miRNA dysregulation has been shown during HIV infection and is also involved in breaches in the gastrointestinal integrity found in HIV-infected individuals (81). Nonetheless, we did not find these affected in our system, and for the miRNAs with significant fold change in our system, the functions remain unknown. Potentially, they might be involved in limited inflammation and antiviral responses as well as increased HIV infection, seeing their important role in regulating responses, seen in our complement-opsonized HIV- exposed tissues and hence needs further investigation.

One limitation of the study is the use of spinoculation to infect the mucosal tissue (37) seeing that this method allows the virions access to the tissue from more than the apical/epithelial side. The rational for using this method was that it achieves a high level of HIV-1 infection compared to other infection methods.

In conclusion, we observed enhanced HIV infection in the cervical tissue when the virions were opsonized with complement and/or immunoglobulins. Pathway analysis of cervical tissue gene expression showed enrichment of factors related to the upregulation of different immune modulatory and inflammatory pathways that were time dependent. Proteomics showed that most of the immune pathway genes activated on transcriptional level did not translate into proteins. We found evidence of active suppression of immune responses, which offers an environment advantageous for HIV infection. Complement opsonization is critical in the early events of sexual transmission of HIV infection. The effects exerted by the complement-opsonized virions should be considered in the design of drugs and strategies to prevent HIV transmission.

## Data Availability Statement

The data presented in the study are deposited in the NCBI GEO repository, accession number GSE160774.

## Ethics Statement

The study was approved by the Linköping University Ethical Review Board (Ethical permit EPN M206-06). Written informed consent for participation was not required for this study in accordance with the national legislation and the institutional requirements.

## Author Contributions

CS, RE, MK, EC, MS, SN, ML, ES, KB, and AB performed experiment and/or analyzed data., NB provided human cervical tissue samples. CS, RE, SN, ES, and ML wrote the manuscript. NB, KB, EC, MK, and AB edited the manuscript. All authors contributed to the article and approved the submitted version.

## Funding

This work was supported in part with Federal funds from the National Cancer Institute, National Institutes of Health, under Contract No. HHSN261200800001E.

## Conflict of Interest

The authors declare that the research was conducted in the absence of any commercial or financial relationships that could be construed as a potential conflict of interest.
