# Corrigendum: Complement-Opsonized HIV Modulates Pathways Involved in Infection of Cervical Mucosal Tissues: A Transcriptomic and Proteomic Study

**DOI:** 10.3389/fimmu.2021.730130

**Published:** 2021-07-08

**Authors:** Cecilia Svanberg, Rada Ellegård, Elisa Crisci, Mohammad Khalid, Ninnie Borendal Wodlin, Maria Svenvik, Sofia Nyström, Kenzie Birse, Adam Burgener, Esaki M. Shankar, Marie Larsson

**Affiliations:** ^1^ Division of Molecular Medicine and Virology, Department of Biomedicine and Clinical Sciences, Linköping University, Linköping, Sweden; ^2^ Department of Obstetrics and Gynecology, University Hospital, Linköping, Sweden; ^3^ Department of Obstetrics and Gynecology, Region Kalmar County, Kalmar, Sweden, and Department of Biomedical and Clinical Sciences, Linköping University, Linköping, Sweden; ^4^ Department of Clinical Immunology and Transfusion Medicine, and Department of Biomedical and Clinical Sciences, Linköping University, Linköping, Sweden; ^5^ National HIV and Retrovirology Labs, JC Wilt Infectious Disease Research Centre, Public Health Agency of Canada, Winnipeg, MB, Canada; ^6^ Center for Global Health and Diseases, School of Medicine, Case Western Reserve University, Cleveland, OH, United States; ^7^ Infection Biology, Department of Life Sciences, Central University of Tamil Nadu, Thiruvarur, India

**Keywords:** cervical tissue, complement opsonized HIV-1, innate immunity, primary infection, proteomics, transcriptomics, HIV-human immunodeficiency virus

## Incorrect Affiliation

In the published article, there was an error in affiliation 3. Instead of “Women’s Clinic, Länssjukhuset, Kalmar, Sweden”, it should be “Department of Obstetrics and Gynecology, Region Kalmar County, Kalmar, Sweden, and Department of Biomedical and Clinical Sciences, Linköping University, Linköping, Sweden”.

The authors apologize for this error and state that this does not change the scientific conclusions of the article in any way. The original article has been updated.

