# Supplementary material for: Complement-Opsonized HIV Modulates Pathways Involved in Infection of Cervical Mucosal Tissues: A Transcriptomic and Proteomic Study
Source: Front Immunol. 2021 May 20;12:625649. doi: 10.3389/fimmu.2021.625649 (PMC8173031; doi:10.3389/fimmu.2021.625649)
Supplement: Supplementary file 1 [file DataSheet_1.pdf]

Supplementary Figures Svanberg et al

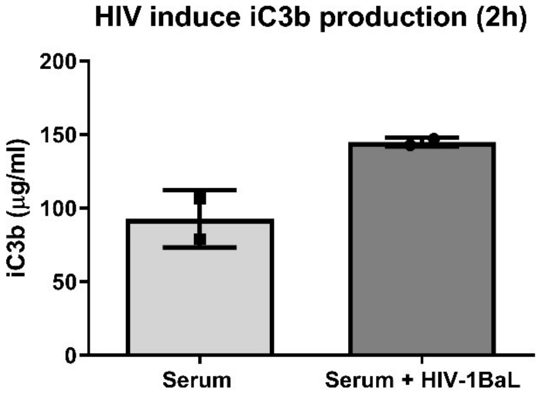

Supplementary Figure 1. HIV activate the complement cascade leading to iC3b production.

HIV was incubated for 2h with an equal volume of serum, thereafter, spun down using an ultracentrifuge and washed before being analyzed using an iC3b ELISA.

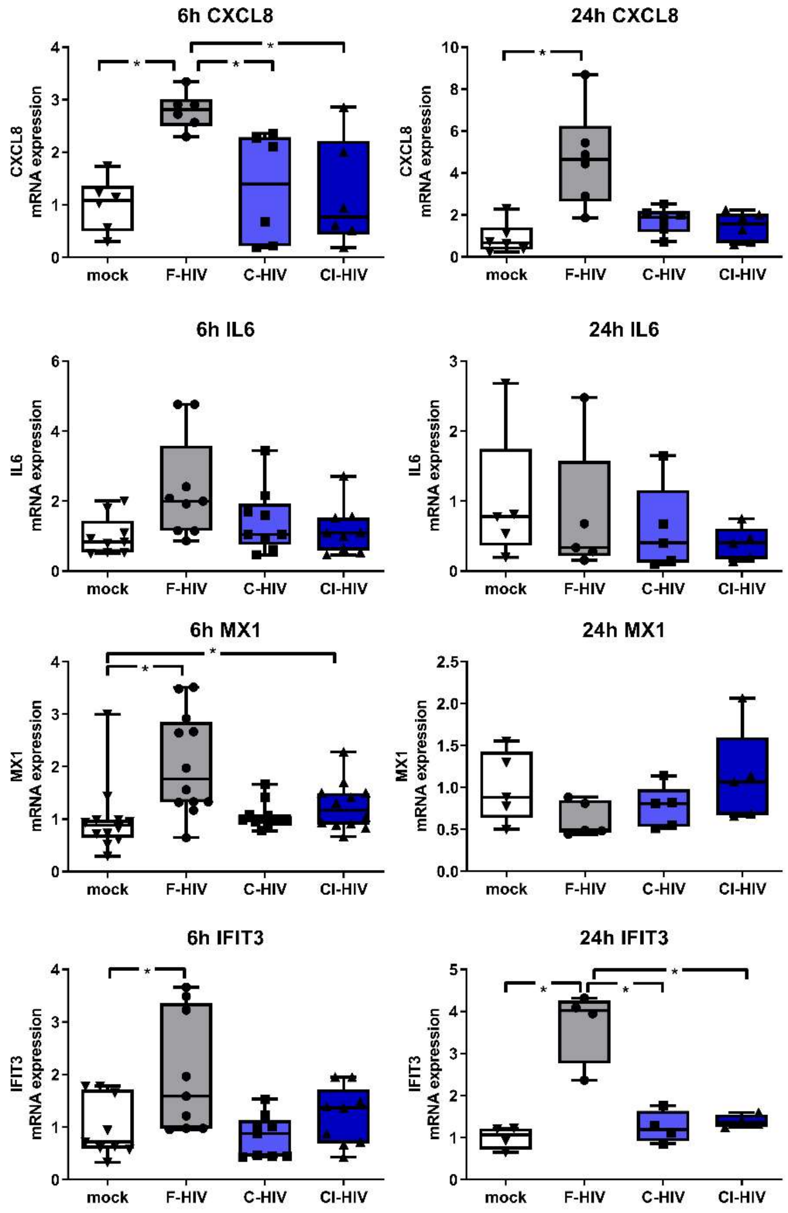

Supplementary Figure 2. Free HIV induce transcription of factors involved in inflammation and interferon signaling.

Cervical mucosal biopsies (3mm) were spin-occulated with 250ng/ml F-HIV, C-HIV; CI-HIV or mock for 2 hours. The biopsies were then washed and incubated for an additional 4h or 22h. The tissues were harvested, and RNA was purified, and qPCR was performed. The mRNA levels of the inflammatory markers CXCL8 and IL6 and the interferon induced genes MX1 and IFIT3 was measured. *$p<0.05$, **$p<0.01$

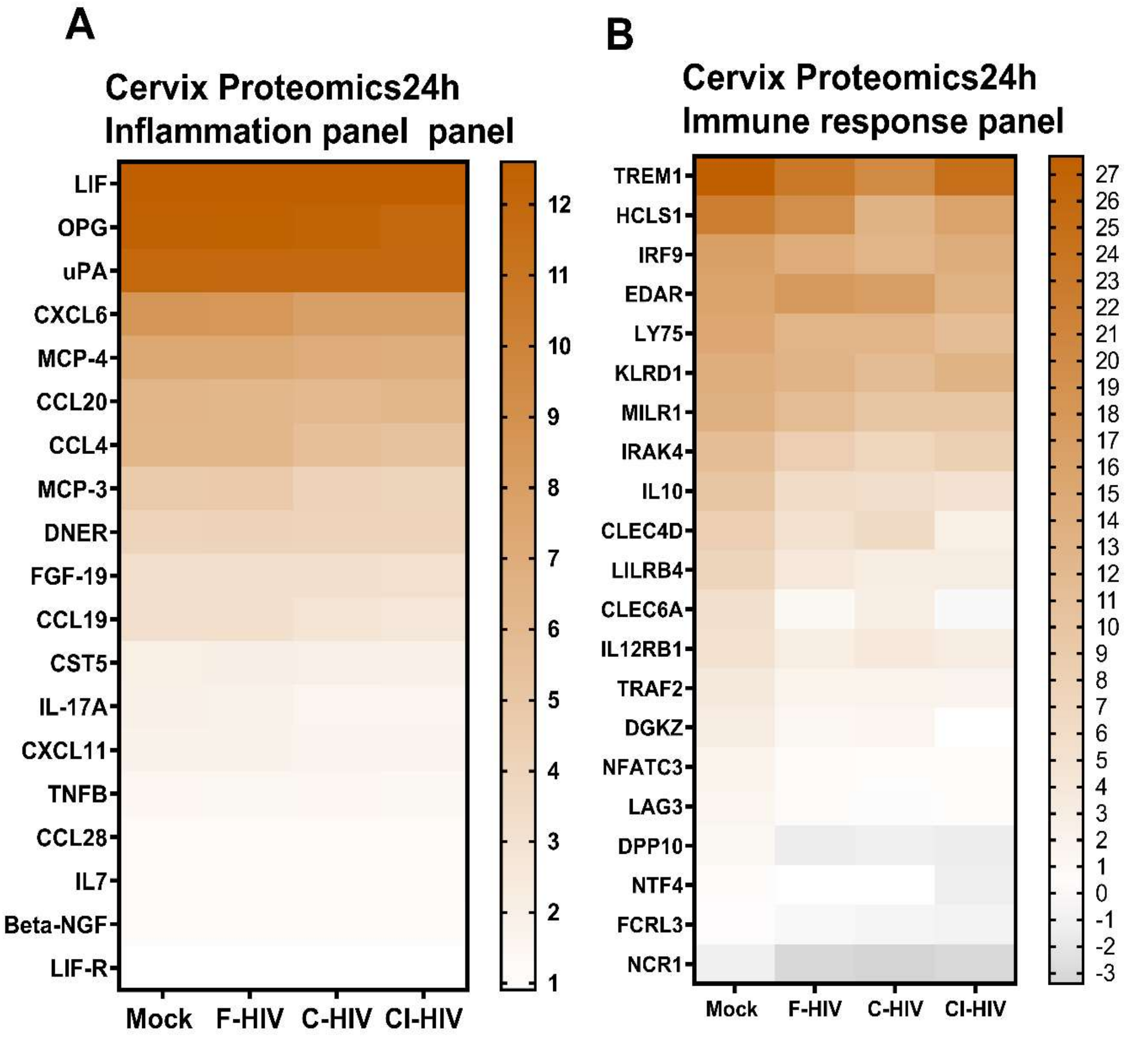

Supplementary Figure 3. The proteomic profiles of inflammatory and immune response factors indicate a low effect of the HIV exposure with a trend of suppressed protein expression.

Cervical mucosal biopsies (3mm) were spin-occulated with 250ng/ml F-HIV, C-HIV; CI-HIV or mock for 2 hours. The biopsies were then washed and incubated for an additional 22h. The supernatants were harvested analyzed with Proseek 130 ® multiplex proteomics immunoassay (Olink Proteomics, Uppsala, Sweden) with the Inflammation and Immune Response proteomics panels. Included in the heat maps are proteins passing quality control, with minimum one of the F-HIV, C-HIV, or CI-HIV groups with significant changes compared to mock.
